# Multidimensional and Intersectional Gender Identity and Sexual Attraction Patterns of Adolescents for Quantitative Research

**DOI:** 10.3389/fpsyg.2021.697373

**Published:** 2021-09-17

**Authors:** Wassilis Kassis, Dilan Aksoy, Céline A. Favre, Sibylle T.-G. Artz

**Affiliations:** ^1^Department of Research & Development, School of Education, University of Applied Sciences and Arts Northwestern Switzerland, Windisch, Switzerland; ^2^School of Child and Youth Care, Faculty of Human and Social Development, University of Victoria, Victoria, BC, Canada

**Keywords:** gender identity, intersectionality, gender diversity, gender pattern, gender diverse youth

## Abstract

To identify and compare gender identity and sexual attraction (GISA) patterns using a latent class analysis (LCA), questionnaire data from a cross-sectional study on social resilience in adolescence was conducted in 2020, using a sample of 785 Swiss seventh grade high school students. Following McCall’s complex intersectionality approach, we applied an intracategorical and intersectional approach to reshape, differentiate, and critique the existing binary, heteronormative GISA categorization. To empirically validate the detected classes according to content, we measured the participants’ psychological characteristics with measures of self-esteem, social competence, symptoms of anxiety and depression, dissociation, social desirability, and emotional styles, and related these measures to the respective GISA patterns the LCA detected. The results of our multistep LCA endorsed that heteronormatively binary gender identities are far too simplistic to fully illustrate adolescents’ differences and similarities where gender is concerned. Out of the subsample of *n* = 785 adolescents (375 identified as “assigned females” and 410 “assigned males”), three significant subgroups of multidimensional GISA patterns emerged for both assigned females and males where differences within the identified GISA groups were larger than those between traditional “boys” and “girls” overall. The LCA demonstrated that the six classes with GISA indicators could be described as low GISA diverse (*cis*/heterosexual), intermediate GISA diverse (gender identity diverse and/or sexual diverse), high GISA diverse (gender diverse/sexual diverse) for both assigned males and females thus showing that GISA and the psychological state according to gender variance is greater within groups of assigned females and assigned males than between these groups.

## Introduction

In questioning the way indicators for gender identity and sexual attraction (GISA) (see the section “Glossary”) are applied in social sciences research, we consider not only the empirical validation of multidimensional GISA categories, but also the theoretical frameworks that help us to understand the categories that are applied to gender, gender identity and sexual attraction. Additionally, when reshaping GISA categories and testing for gender diversity, we focus mainly on identifying and gaining access to existing social identities that are already in practice, but thus far are not visible because of the still dominant empirical binary understanding of gender. Our intent is to readjust empirical binary gender categories to gain a more valid picture of GISA in adolescence, while knowing that we are only at the beginning of developing a more heterogeneous representation of social identities in social sciences.

Overwhelmingly, in most research areas, gender serves as an unquestioned dichotomous and distinct socio-demographic indicator covered by a single item on a questionnaire dividing a sample into males and females. This not only discriminates against individuals who identify themselves outside of this binary categorization, but also leads to biased research findings (Cameron and Stinson, 2019). At the same time, following the intrasectional intersectional perspective of McCall (2005) and considering categorization to be *the* precondition for statistical analyses in quantitative research (Frohard-Dourlent, 2016), gender identity self-identification is a demanding and unanimously performed mission (Lindqvist et al., 2020). Based on Butler’s (1999a) concept of “gender performativity,” gender identity and sexual identity are connected as ongoing sequences of acts and patterns, and gender and sexuality are placed in the context of social power discourses. From this, it can be gathered, in line with other studies (Eisenberg et al., 2019), that while gender identity exists independently of sexual attraction (see the section “Glossary”), the two social identities (gender identity and sexual attraction) are intersectionally related. When it comes to studying gender self-identification, the dynamic gender identity interactions between an individual’s acts and contextual power patterns are always a current state connected to each person’s respective developmental stage and that of with whom they share their life worlds. This is especially, but not only (Tate et al., 2014), interesting in adolescence because of the rapid and crucial changes connected to gender identity during that time (Diamond and Butterworth, 2008; Steensma et al., 2013; Dembroff, 2019).

Based on Butler’s (1999a) concept of “gender performativity,” we envision gender identity and sexual identity as ongoing sequences of acts and patterns and we place gender and sexuality in the context of social power discourses. In doing this, we accept that the interpretation and performativity of “body” cannot exist outside a gendered discourse, which, of course, does not mean that there is no such thing as the material body (Blumenfeld, 2017). Especially when challenging gender identity categorization, there needs to be an awareness of the differences between gender diversity (see the section “Glossary”) as the level to which a person’s gender identity differs from the cultural norms prescribed for people of a particular assigned sex (see the section “Glossary”) (Institute of Medicine, 2011) and the challenging term “gender dysphoria.” The second term is typically used to diagnose a mental disorder according to the *Diagnostic and Statistical Manual of Mental Disorders* (5th ed.; *DSM-5*) and the International Classification of Diseases (ICD) (Fraser, 2015; Poteat et al., 2019) based on a discrepancy between a person’s gender identity and assigned sex that causes clinically significant distress or impairment (Knudson et al., 2010).

Understanding the societal power debates (Butler, 2004; Devor, 2007), the discussion on gender terminology, and the empirical validity of gender variety in adolescence is crucial to positively supporting adolescents’ lives (Coleman et al., 2012; Boskey, 2014). Not only the plethora of terms (Lau et al., 2020), but also the very dynamic and context-specific discussions about the respective terminology can be overwhelming (Vance and Mesheriakova, 2017), even if the relevant empirical validation of the categories that are being discussed is mostly poor. In adolescence, sexual and gender minorities are among the most marginalized and least surveyed groups in the social sciences (Stieglitz, 2010; Clark et al., 2014), even while the barriers that diverse (see the section “Glossary”) adolescents experience lead to higher levels of stigma (Pardo and Devor, 2017) and to social (Clark et al., 2014) and societal barriers (Roberts and Fantz, 2014). The complexity of gender, gender identities, and psychological (dis-)comfort with gender typicality has been an important topic for a few decades now, largely evolving from two perspectives. Firstly, the political and deconstructive perspective, which is grounded in feminist and queer theoretical work and aims to identify hidden normative values and power struggles that emerge within gendered societies (see de Beauvoir, 1953; Friedan, 1963; Butler, 1990; Herdt, 1996). Richards et al. (2016) reviewed the limited research in this field and emphasized that, non-binary people might appear to be a relatively recent and under-researched phenomenon, but it is likely that some people who previously would have identified as trans(sexual) – that is within the gender binary, but moving across it – may have identified outside of the binary if that discourse had been available to them (p. 89).

Thus, they proposed to diversify gender based on the concept of a continuum (Richards et al., 2016), which is increasingly in accordance with biologists’ conception of gender (Ainsworth, 2015; Zhao, 2018). According to Westbrook and Saperstein (2015), research defines how society understands structural constructs, which makes it important for academics to rethink gender, gender identity and sex in ways that move us beyond the commonly used binary concepts so that we may have a better understanding of gender processes and reduce inequality in power relations. Although research has shown that the gender binary is inadequate, “difference phenomena” such as depression prevalence are still being explained in terms of heteronormative gender differences without an understanding of the underlying mechanisms (Hyde et al., 2019).

Secondly, the perspective that has emerged from empirical research concerning children’s and adolescents’ gender identity development (e.g., Egan and Perry, 2001; Martin et al., 2002; Tobin et al., 2010), which conceptualizes gender identity as multidimensional, and not “just” more fluid than the currently used binary categorization suggests. This multidimensional perspective is also interested in analyzing the negative psychosocial outcomes (e.g., low self-esteem and life satisfaction, poor mental health from high levels of anxiety and depression, and suicidal thoughts and attempts) that can be noted when children are not permitted to freely express their gender identity in important developmental contexts such as their homes; with siblings, parents, caretakers and other family members; and in their schools, with teachers and school mates and with other people whom they also encounter in these contexts (Newhook et al., 2018).

In recent years, the gender binary has been challenged from the viewpoint of different disciplines such as neuroscience, in investigating the human brain; behavioral endocrinology, in challenging the “dimorphic hormonal systems”; psychology, in highlighting similarities between assigned males and females as well as trans and non-binary identities; and developmental research, in looking at the binary gender identity concepts as culturally determined (see Hyde et al., 2019). Further, as Cole (2009) stated, “attending to who is included within a category can lead to a more nuanced understanding of how social categories of identity, difference, and disadvantage shape experience” (p. 173). Interestingly, despite the acknowledged need to move beyond binary concepts, and the developments in a number of fields briefly noted here, empirical tools involving diverse gender identity scales are still not the norm. For these reasons we aim to create a measure of gender identity that contains both gender identity and sexual attraction identification for adolescents that is multidimensional, intersectional and includes GISA on a continuum, while also considering the gendered social context in which 12-year-olds live. This study addresses four research questions: (1) Are there distinct intracategorical intersectional GISA patterns? (2) What levels of the introduced psychological variables are associated with the identified GISA patterns? (3) How similar are the identified GISA patterns with their corresponding psychological variables for both assigned males and females?” and (4) How different will the psychological picture of the new GISA patterns be when comparing to the psychological picture of the heteronormative categories of “girls” and “boys.”

### Identifying Gender Identity Empirically

Thus far, gender research seems to have been developing in two mostly independent research areas: One area looks at a multidimensional gender identity consisting of several factors and takes place mostly in psychological development research (Ruble et al., 2006; Steensma et al., 2013; Roberts and Fantz, 2014; Perry et al., 2019). Another area emerges primarily from queer and health research and looks at non-binary gender identities mostly focused on trans identities (Butler, 2009; Westbrook and Saperstein, 2015; Skinner et al., 2018; Poteat et al., 2019), although methodology and research contents on non-binary adolescents are still limited (Scandurra et al., 2019).

The conceptualization of gender identity from a solely biological perspective seen as playing a dominant role in determining women’s and men’s social identities has been thoroughly critiqued and analyzed by scholars involved in the second-wave feminist movement (see de Beauvoir, 1953; Friedan, 1963). As well, in Money and Ehrhardt (1972), proposed a theory — which was provocative at that time – that posited that social factors dominated biological factors where gender identity and gender roles were concerned, and in Maccoby and Jacklin (1974), were able to empirically show that within-gender differences were greater than between-gender differences. Additionally, Bem (1974) was one of the first challengers of the unidimensional understanding of gender identity by showing that both women and men possess feminine and masculine characteristics (Zosuls et al., 2011). Bem (1974) also noted that because of this, femininity and masculinity should be treated as two independent dimensions that could be found in both women and men. The multidimensional understanding of gender identity development was then further refined by multiple researchers focusing on different aspects of this, such as sex typing (Huston, 1983), the role of gender stereotypes (Martin et al., 2002; Ruble et al., 2006), and the gender self-socialization model (Tobin et al., 2010).

Judith Butler (1990) provided an additional direction as she initiated a paradigm shift in feminist research in stating that not only gender but “sex” was also socially constructed. This led queer theorists to deconstruct sex and gender categories and question the hidden normative values and power struggles that, to this day, are embedded in these categories (Babka and Posselt, 2016). Further, in the *DSM-5*, the *DSM IV-TR* (American Psychiatric Association, 2000), the diagnosis of gender identity disorder, which only recognized gender in male–female binary terms and described wishing to change one’s gender to be a disorder, was removed (Richards et al., 2016) and replaced with the term “gender dysphoria” (Lev, 2013). Accompanying this change is an ongoing debate about summarizing the emotional stress experienced by gender diverse people under the term gender dysphoria (Meyer-Bahlburg, 2010; Lev, 2013). Meyer-Bahlburg (2010) concluded his extensive investigation of this debate by pointing out that “gender incongruence” would be a more suitable term to explain “the incongruence of one’s gender experience and expression with one’s assigned sex and, where applicable, one’s congenital primary and secondary sex characteristics” (p. 471).

Furthermore, for children and adolescents formulating their senses of self and others, it is especially vital to identify and connect with their own gender identity. Because of the societal power relations connected to GISA, persistent discomfort with socially expected gender typicality creates impediments for adolescents’ emotional and social development (Hepp et al., 2005; Rieber, 2006; Diamond, 2013), and transgender adolescents have been shown to be bullied and to perpetrate bullying at elevated rates (Heino et al., 2021). As well, gender diverse adolescents’ self-concept (e.g., self-esteem), emotional stability (e.g., emotional styles, dissociation, anxiety, and depression), and positive social relations to their peers (e.g., social competence and social desirability), which are crucial for their development, are identified as under pressure and subjected to special health care treatments (Baams, 2018) while the societal power relations leading to exactly these symptoms (Hoshiai et al., 2010) are mostly not addressed (Eisenberg et al., 2017).

### Gender Identity From a Psychological Perspective

Developmental psychologies have thus far focused on studying the effect of gender knowledge on social competence and emotional styles (Menon and Gorman, 2019). Following, and building on her Bem (1974; 1981; 1984) work, which revised the predominantly used unifactorial masculinity–femininity continuum in developmental psychology that stated that femininity and masculinity should be treated as two independent dimensions found in both women and men, Spence (1993) developed a multifactorial gender identity theory. Spence showed that “sex typing” was multidimensional and therefore typical gender behavior should be assessed on different dimensions. Egan and Perry (2001) built on Spence’s (1993) “absence of an operational definition for her construct of summary self-perceived gender typicality” (p. 452) that relied on self-ratings of the adjectives feminine and masculine. However, Egan and Perry (2001) also found a limitation in Spence’s approach and therefore noted that while she suggested that in general, gender typicality could be captured through self-ratings classified as *feminine* and *masculine*, she also pointed out that feelings about gender typicality, which these self-ratings garnered, were influenced overall by “other factors such as their knowledge of their biological sex” and therefore “self-ratings on these adjectives cannot be interpreted unambiguously as indexes of self-perceived gender typicality” (p. 452).

Egan and Perry (2001) therefore proposed measuring self-categorization of gender identity using four dimensions:

(a) knowledge of membership in a gender category, (b) felt compatibility with one’s gender group (i.e., self-perceptions of gender typicality as well as feelings of contentment with one’s gender), (c) felt pressure for gender conformity, and (d) attitudes toward gender groups (p. 451).

In conceptualizing gender identity multidimensionally in ways that could be employed in both an enhanced theoretical and conceptual understanding of gender and in empirical research, Egan and Perry (2001) showed that gender identity was a complex social concept that should and could not be measured unidimensionally and that general self-declarations of femininity or masculinity as a form of gender typification were not enough because many factors influenced these multidimensional categories (Egan and Perry, 2001; Perry and Pauletti, 2011). With that, they showed that gender diversity on its own was not inherently distressing, and that it was the perceived pressure for gender conformity from others in one’s lifeworld that could result in gender-related distress (Hegarty, 2009). Although Egan and Perry (2001) laid important groundwork for measuring gender identity multidimensionally and empirically, other authors have critically examined and modified some aspects of their model. Firstly, although the measurement tool Egan and Perry (2001) developed has been used and adapted by various researchers (see, e.g., Leaper et al., 2012; Kornienko et al., 2016; Hoffman et al., 2019), but still retains a binary understanding of gender identity in that survey participants’ responses are categorized and analyzed according to the gender/sex that was assigned to them at birth. As Hoffman et al. (2019) stated: “As in Egan and Perry’s (2001) measure, both gender typicality and felt pressure measures included items that were gender specific; thus, girls and boys completed separate versions” (p. 311).

As well, while Egan and Perry offered a multidimensional approach to measuring gender identity, they also stated that “By age 6 or 7, nearly all children attain full gender constancy, thereby eliminating within-sex variability on this facet of gender identity. This fact means that beyond this age, this aspect of gender identity cannot account for within-sex individual differences in other variables, such as sex typing or adjustment” (p. 451).

This shows that for these researchers, gender membership is a fixed category known to most children by the age of 6 or 7, which makes gender membership immutable in these researcher’s eyes, thereby eliminating both the possibility of and the need for examining within-gender category gender differences. In keeping with Egan and Perry’s earlier claims, in their more recent review of the research on gender identity in school children, Perry et al. (2019), also noted that they found hardly any differences or variations in within gender self-categorization. Yet in contrast to Perry and Egan’s earlier work, Perry et al.’s (2019) more recent ongoing research in the genderqueer context (an umbrella term for gender identities outside the binary axis), had for some time quite convincingly shown that knowledge of membership in a gender category (gender self-categorization) was a dynamic process that could greatly change over time between and beyond genders (Diamond and Butterworth, 2008; Dembroff, 2019).

Further to this, the gender typing scale, which Egan and Perry (2001) originally developed, has been revised and applied in a number of more recent studies because the original scale has a complex question format (Leaper et al., 2012; Kornienko et al., 2016; Hoffman et al., 2019). In this regard, Martin et al. (2017) have suggested a dual-identity approach that entails comparing oneself to two gender groups (i.e., to own-gender and other-gender individuals) and therefore allows a broader spectrum of gender identities because individuals can identify with two overlapping dimensions. As well, before Martin et al. (2017); Horn (2008) noted that feelings of “gender atypicality” (low own-gender typicality) are related to poor outcomes such as lower levels of social competence and peer exclusion, and could lead, as Carver et al. (2003) showed, to feelings of low self-esteem and greater risk for anxiety, depression, and suicide. This also holds for higher levels of dissociation experienced as a disruption or discontinuity of consciousness (Colizzi et al., 2015) for gender-atypical adolescents. Interestingly, however, the findings in this research considered atypicality only in terms one’s own gender. In relation to this, Martin et al. (2017) showed, with their dual-identity approach, that when comparing themselves to their own-gender and other-gender individuals, children with low typicality have low feelings of belonging, whereas children with high typicality with both their own and the other gender tend not to. These insights indicate that feeling similar to the own-gender group provides a strong foundation that supports and relates to good adjustment, whereas experiencing other-gender typicality offers additional adjustment-related benefits through sense of relatedness to both gender groups (Martin et al., 2017). These feelings of gender similarity and gender typicality with the own-gender group and the reassurance of being in a majority group are connected with adolescents’ higher levels of emotional stability (Hepp et al., 2005), leading to positive emotional self-awareness, social sensitivity to others’ emotions, and to lower levels of disrupted consciousness such as dissociation (Rieber, 2006).

These findings strongly suggest that experiencing persistent discomfort with expected gender typicality is a well-identified risk factor where adolescents’ emotional and social development (Hepp et al., 2005; Diamond, 2013) is concerned. So, while felt same-gender typicality is rarely associated with negative outcomes, though sporadically it is associated with aggression in children of both sexes (Yunger et al., 2004; Pauletti et al., 2014), gender non-typical adolescents would be expected to have higher levels of negative developmental outcomes.

### Intersectionality and Gender Identity and Sexual Attraction Patterns

In using the intersectionality framework originally developed by Crenshaw (1994) to describe the intersections of social identities of gender and race, and for analyzing multiple social identities such as age, gender identity and sexual attraction (Eisenberg et al., 2019), we acknowledge there is no position of implied freedom beyond discourse and recognize that gender identity is constrained by the power structures within which it is located. We also acknowledge that although age, gender identity and sexual attraction are three distinctly separate identities it is important to look at these from an intersectional point of view in order to identify hidden and openly celebrated power structures. Thus, following McCall’s (2005) complex intersectionality approach, there are three possible ways to apply intersectionality: first, the anticategorical intersectional approach, which rejects current categories; second, the intracategorical intersectional approach, which critiques and reshapes current categories; and third, the intercategorical intersectional approach, which combines and reorders existing categories. The intercategorical intersectional approach is very often wrongly seen as *the* intersectional approach and stands regularly *pars pro toto* as a synonym for intersectionality. It is applied mostly in quantitative research and is sometimes used solely as an analytical toolkit for a more concise and stronger prediction of the respective models to be estimated while skipping the political notion of addressing power relations (Westbrook and Saperstein, 2015).

The intracategorical intersectional approach reshapes while differentiating and critiquing essentialist social categories, and is used in qualitative and quantitative research. It asks how categories can be applied in more complex ways and introduces a thorough examination of the power relations embedded in scientific theoretical and methodological approaches that are employed when constructing social categories. Not contrary to but distinct from the anticategorical approach, it acknowledges the very existence of the social categories that are to be differentiated and focuses on reducing power relations that scientific methodology expresses (Saperstein, 2012).

To reflect the social power discourses that are the contexts for gender, we applied an intracategorical and intersectional approach to develop multifaceted and complex GISA patterns for quantitative research. In doing this we recognized from the outset that from a psychometric point of view, it was indeed challenging to pursue an intracategorical approach with a sample consisting of adolescents who were between 12 and 13 years old at the first measurement point because they were in a developmental stage full of changes (e.g., Kornienko et al., 2016). With this, we also recognized the challenge of conceptualizing GISA in a way that did justice to theoretical concepts that could simultaneously be compactly applied.

As our exploratory approach is not driven by hypotheses, following McCall’s (2005) intersectionality approach, we applied an intracategorical and intersectional approach to reshape, differentiate, and critique the existing binary, heteronormative GISA categorization that hinders autonomous development and free choice. The complexity of sexual attraction, gender identities, and psychological (dis-)comfort with gender typicality was tested empirically. Additionally, in order to empirically validate the latent gender classes according to content, we measured the participants’ psychological characteristics with measures of self-esteem, social competence, symptoms of anxiety and depression, dissociation, social desirability, and emotional styles.

## Materials and Methods

### Study and Sample Descriptives

The data we analyzed came from a cross-sectional sample (to be followed by additional three survey-waves within the next 3 years) of a broader study on adolescents’ violence resilience despite experiencing family violence, conducted in the autumn of 2020. The random sample consisted of 785 seventh grade high school students from Switzerland, 375 (47.8%) assigned females and 410 (52.2%) assigned males who anonymously completed an online questionnaire. Consent forms were obtained from students and their caregivers. No incentives were given. The research ethics committee at the University in Zurich, Switzerland, authorized the project. On the day of the study, the research team members gave a short oral introduction about the study to the students who were present in the participating 49 classes and then these students completed the questionnaire in about 60 min. The overall sample average age was *M* = 12.9 (*SD* = 0.71). Of the participating students 491 (62.5%) were Swiss citizens.

### Measures

To gather our data, we employed three measures for gender identity, one for sexual attraction and seven measures for psychosocial factors.

#### The Three Gender Identity Measures and Sexual Attraction

To create our three measures of gender identity we adapted and revised Egan and Perry’s (2001) scales based on current research on gender identity self-assessment and applied an intracategorical approach as described below:

##### Gender identity diversity

Gender identity diversity refers to the respective adolescents’ knowledge of membership in a gender category and builds on Egan and Perry’s (2001) gender categorization. As we accept gender is on a continuum, the term “knowledge about gender category membership” is insufficient to describe the gender by which an individual wishes to be known (City of Portland, 2020); therefore, we asked adolescent respondents where they recognized themselves on a six-point continuum, single question item: “1 = clearly like a girl,” “2 = more like a girl,” “3 = more like a boy,” and “4 = clearly like a boy,” with the possibility of choosing “5 = both” as well as “6 = none.” Further, since the terms “female” and “male” are often associated with one’s assigned sex, we used “girl” and “boy” as the preferred terms to assess gender identity (Tate et al., 2013; Ansara and Hegarty, 2014). Although we used “girl” and “boy” as the opposite of each pole on the gender continuum, adolescents could choose an intergender-overlap (“more like a …” or “both”), as well as the option “none.” So far, research concerning multidimensional gender identity has neglected this dimension. The response options were categorized into a dichotomous variable with the label 0 as “individual gender identity knowledge corresponds with assigned sex” (i.e., cisgender) and label 1 as “individual gender identity knowledge counteracts assigned sex” (i.e., gender diverse). For assigned females, label 0 covered responses “1 = clearly like a girl” or “2 = more like a girl,” and label 1 responses “3 = more like a boy,” “4 = clearly like a boy,” “5 = both,” or “6 = none.” For assigned males, label 0 was covered responses “3 = more like a boy,” “4 = clearly like a boy,” and label 1 responses “1 = clearly like a girl,” “2 = more like a girl,” “5 = both,” or “6 = none.”

##### Gender typicality at school

Starting from Egan and Perry’s (2001) concept of gender typicality, we focused on gender identity expressions in different social contexts and thereby tested the participating adolescents’ respective gender typicality in specific social contexts because gender identity expressions are always socially dependent. As previously stated, gender identity expressions are multidimensional constructs and therefore should not be measured on a single dimension (Egan and Perry, 2001; Perry and Pauletti, 2011). We therefore firstly related the question about gender identity expression to the school context (Jewell and Brown, 2014), and secondly, following Martin et al. (2017), we measured gender identity expressions with an adapted five-item version of Egan and Perry’s (2001) six-item instrument. Before us, Martin et al. (2017) followed a dual-identity approach, asking assigned females and males separately about their own-and-other gender typicality to describe gender performativity. We expanded Martin et al.’s (2017) dual gender approach that was constructed for children by using a continuous understanding of gender identity. Therefore, we applied a continuum answer structure, that is, a Likert scale, for self-affirmed gender typicality (i.e., clearly like girls, more like girls, both, more like boys, clearly like boys, none), and we posed the same questions to all participating adolescents. We also modified Martin et al.’s (2017) last item from “how much time do you like to spend with boys/girls” to “how do you like to spend your school break time.” Further, we adapted the English version of the questions to the Swiss-German school context using language that young adolescents would understand. The response options were categorized into a dichotomous variable with the label 0 as “gender typicality at school corresponds with social heteronormative expectations” and label 1 as “gender typicality at school conflicts with social heteronormative expectations.” For assigned females, label 0 covered on all five questions responses “1 = clearly like girls” or “2 = more like girls,” label 1 covered on all five questions responses “3 = both,” “4 = more like boys,” “5 = clearly like boys” or “6 = none.” For assigned males, label 0 covered on all five questions responses “4 = clearly like boys” or “5 = more like boys,” label 1 covered on all five questions responses “1 = more like girls,” “2 = clearly like girls,” “3 = both” or “6 = none.” Because of our LCA-approach, only dichotomous categorizations were possible. Therefore, when at least one question was labeled as 1 “gender typicality at school conflicts with social heteronormative expectations,” the overall *Gender Typicality at school* indicator was labeled as 1 “gender typicality at school conflicts with social heteronormative expectations.”

##### GISA expectations

To measure respondents’ *GISA expectations*, we developed a new indicator by two answers-sets to determine adolescent participants’ contentment and pressure concerning (a) gender identity expectations and (b) sexual attraction. Therefore, we asked the following questions: “Are there people in your life who want you to be different?” To provide their answers, after the statement “I think that I should,” respondents were presented with two sets of questions with four possible answers: One set of answers focused (a) on gender expectation (“be more like a girl,” “be more like a boy,” “be as I am,” and “don’t know”), and the other set of questions focused (b) on sexual attraction expectation (“be romantically/sexually interested in girls,” “be romantically/sexually interested in boys,” “be as I am,” and “don’t know”). The response options were categorized into a dichotomous variable with the label 0 as “self-expectations fulfilled social heteronormative expectations” and label 1 as “self-expectations did not fulfill social heteronormative expectations”: For assigned females, label 0 covered on both questions, so for (a) and (b), responses “3 = be as I am” or “4 = don’t know,” and the following responses on the questions focused on sexual attraction (b) “2 = be romantically/sexually interested in boys,” and “3 = be as I am,” as well as “4 = don’t know.” For assigned males, label 0 covered on both questions,’ so for (a) and (b), responses “3 = be as I am” or “4 = don’t know,” and the following responses on the questions (b) focused on sexual attraction “1 = be romantically/sexually interested in girls,” and “3 = be as I am,” as well as “4 = don’t know.” When at least one of the two answers-sets (a or b) was labeled as 1 “self-expectations did not fulfill social heteronormative expectations,” the overall GISA expectations indicator was labeled as 1 “self-expectations did not fulfill social heteronormative expectations.”

##### Sexual attraction

Following Collier et al. (2013), as a measure for *sexual attraction*, the respondents were asked: “Who are you romantically or sexually attracted to?” on two five-point scales that allowed participants select to “to girls” and “to boys,” according to the options of “1 = no,” “2 = probably no,” “3 = sometimes,” “4 = probably yes,” and “5 = yes.” Thus, the respondents were offered a range of possibilities for overlap and diversity where sexual attraction was concerned. The response options were categorized into a dichotomous variable with the label 0 as “heterosexual” and label 1 as “sexual diverse.” Label 0 covered responses “3 = sometimes,” “4 = probably yes,” and “5 = yes” when addressed to a different assigned sex; and the responses “1 = no” and “2 = probably no” when addressed to the same assigned sex. Label 1 covered responses “1 = no” or “2 = probably no” when addressed to a different assigned sex, and the responses “3 = sometimes,” “4 = probably yes,” or “5 = yes” when addressed to the same assigned sex. Label 1 covered also responses “1 = no” and “2 = probably no” when addressed to both sexes.

#### The Seven Psychosocial Factor Measures

##### Symptoms of anxiety and depression

Symptoms of anxiety and depression were assessed through 24 items that were part of the Hopkins Symptom Checklist (Derogatis et al., 1974). From the original 25-item scale version, one item (“*Loss of sexual interest or pleasure*”) was left out because of the participants’ young age of approximately 12–13 years. The items were rated on a four-point Likert scale ranging from 1 = “not at all” to 4 = “extremely,” where higher mean scores indicated a higher severity of anxiety and depression symptoms (Cronbach’s alpha [Cα] = 0.96). Items included were such experiences as “I feel fear” and “Thoughts of ending my life.” For the LCA we performed a median split (MED = 1.56, SD = 0.63), and dichotomized as either (0) lower levels or (1) higher levels on symptoms of anxiety and depression.

##### Self-esteem

Self-esteem was assessed according to the Rosenberg Self-Esteem Scale (Rosenberg, 1965, 2015) for assessing an individual’s global worthiness evaluation. This tool is comprised of a five-item short scale, with higher scores indicating higher self-esteem. The items were rated on a four-point Likert scale ranging from 1 = “not at all” to 4 = “extremely” (Ca = 0.93). Respondents were asked to rate question such as, “In total, I am confident in myself.” We made no adjustments to this scale. For the LCA we performed a median split (MED = 3.00, SD = 0.80), and dichotomized this as either (0) lower levels or (1) higher levels on self-esteem.

##### Social competence with classmates

Anderson-Butcher et al. (2007) originally developed the scale we employed for assessing self-perceived social competence. It measures the ability of becoming positively involved with classmates on five items using a four-point Likert scale (range 1 = “fully disagree” to 4 = “fully agree”) and displayed a high reliability (Ca = 0.92). Respondents were asked to rate questions such as, “I help my classmates.” For the LCA we performed a median split (MED = 3.20, SD = 0.68), and dichotomized this as either (0) lower levels or (1) higher levels on social competence with classmates.

##### Dissociation

The items for assessing dissociation (Colizzi et al., 2015) as a disruption or discontinuity of consciousness were measured on a short scale from an already existing scale, which was used to assess dissociative symptoms (Dissociation Tension Scale acute, DSS-acute). Stiglmayr et al. (2009) developed this scale which consists of one item each on depersonalization (feelings of unreality in relation to oneself), somatoform (sensory and motor disturbances), derealization (feelings of unreality regarding the environment), and analgesia (alterations of sensory processes) that participants could rate on a four-point Likert scale ranging from 1 = “not at all” to 4 = “very much” (Ca = 0.85). For the LCA we performed a median split (MED = 1.00, SD = 0.61), and dichotomized this as either (0) lower levels or (1) higher levels on symptoms of dissociation.

##### Social desirability

The social desirability scale we employed was an adapted short version for adolescents of the KSE-G scale (Kemper et al., 2012), which explores the tendency to give overly positive self-descriptions and asks to what extent participants describe themselves in terms of socially desirable characteristics. The scale we used consisted of three items rated on a five-point Likert scale ranging from 1 = “fully disagree” to 5 = “fully agree” (Ca = 0.82), with a question such as, “Even when I’m stressed, I’m always friendly and polite to others,” with higher scores indicating higher social desirability. For the LCA we performed a median split (MED = 4.00, SD = 0.87), and dichotomized this as either (0) lower levels or (1) higher levels on social desirability.

##### Emotional styles (two scales)

The respondents’ emotional health, that is the emotional profile consistent with study participants’ ways of responding to emotional experiences in their lives (Davidson and Begley, 2013), was captured using the Emotional Styles 24-item questionnaire for adults that Kesebir et al. (2019) we developed and adapted it for adolescents. In our adaptation, we measured two emotional styles: “emotional awareness of one’s emotions” with seven items (Ca = 0.72) and “emotional sensitivity to social context” with six items (Ca = 0.75). All items were rated on a five-point Likert scale ranging from 1 = “disagree” to 5 = “agree.” An example of the questions that were asked about participants’ awareness of their emotions is: “I am not good at identifying my own feelings” (reversed coded). An example of the questions that were asked about participants’ awareness of their emotional sensitivity to social context is: “I have sometimes done things others thought of as tactless or embarrassing” (reversed coded). For the LCA we performed a median split for both scales: Emotional awareness of one’s emotions (MED = 5.14, SD = 0.87), and dichotomized this as either (0) lower levels or (1) higher levels, and the scale emotional sensitivity to social context (MED = 5.33, SD = 0.92), and dichotomized as either (0) lower levels or (1) higher levels.

#### Analytic Strategy

Our analytic strategy aimed to empirically test for adolescents’ multidimensional GISA patterns. First, for the three gender identity dimensions reported (gender identity diversity, gender typicality at school, and gender expectations) and sexual attraction we applied a latent class analysis (LCA) to group participants into empirically distinct GISA “classes” (or patterns) with MPlus Version 8.4. In using invariance analysis with the four dimensions, we ensured reliability for the identified number of patterns (configural invariance) and established the same relevance of the four introduced dimensions (metric invariance).

In the second step, we analyzed the connection between the three empirically validated GISA patterns (see analytic step one) and the respective participating adolescents’ psychological states consisting of seven indicators. This allowed us to employ LCA to determine the significance of these adolescents’ identified multidimensional GISA patterns, given that one of the many possible application fields of LCA is the connection between different pattern sets (Collins and Lanza, 2009), not just the relation between established patterns (e.g., GISA patterns) to specific single variables (Lanza et al., 2013). This complexity was needed because we sought to understand how our established GISA patterns related to a multifaceted psychological state consisting of several psychosocial indicators. This was essential to us, as we needed to validate the identified three GISA classes psychologically and not just treat the psychological variables as covariates to the GISA classes identified. By that, we expected to emphasize the connection to not just a social but also psychological gender as the respective individuals interpreted. To statistically compare the levels of the GISA patterns and psychological dimensions on the identified LCA classes, we ran variance analyses with *post hoc* tests.

### Latent Class Analysis

Latent class analysis (LCA) is a typological rather than dimensional approach. Within one latent class, participants are assumed to have identical patterns of solution probabilities, which means a given item’s solution probability is the same for all individuals belonging to the same class. Because of this individual analysis approach, LCA is designated as a person-centered method. The person-centered methodology notwithstanding, this approach also acknowledges there are differences with respect to the response probabilities possible between patterns. LCA is therefore a statistical method used to empirically classify continuous latent variables (constructs that are not observed directly) from a series of two or more continuous observed variables and to form subgroups of different patterns based on observations that appear to be similar for these subgroups (Hagenaars and McCutcheon, 2002). The observed manifest variables are assumed to be independent of one another once conditioned on the latent variable. This assumption is known as the “local independence” (Hagenaars and McCutcheon, 2002). Thus, individual participants are assigned to the different patterns based on their posterior probabilities for class membership for a particular gender profile. Overall, this methodology allows the grouping of subjects into distinct GISA patterns (classes or groups) according to the indicators reported and included in the analysis, and then it estimates the probability that a particular subject is a member of that class.

Variance analysis with Games–Howell *post hoc* test was performed to determine whether the patterns the LCA identified differed statistically from each other. In conducting a variance analysis, a Welch *F*-Test for unequal variances and additionally the Games–Howell *post hoc* test were applied, because of unequal variances and sample sizes. Variance analysis and Games–Howell *post hoc* tests were applied to identify whether the classes the LCA detected were affected by the different mean levels of the 11 classification indicators, and specifically whether the patterns of more or less heteronormatively gender-typical adolescents showed lower or higher levels of a positive psychological state.

The LCA was conducted for a range of two to five latent GISA classes in the participants. The main aim was to determine if the identified significantly differing classes, that is, the statistically validated GISA patterns, showed a variety within a heteronormative gender categorization that would remain hidden if we stayed with understanding gender as only dichotomous.

The estimated models were non-nested models. Therefore, the procedures chosen for model selection were the sample-adjusted Bayesian information criterion (BIC)^1^ indicating goodness of fit, with a lower value indicating a more appropriate fit, and entropy (Celeux and Soromenho, 1996), indicating the certainty in the estimation, with values above 0.7 considered sufficient (Nylund et al., 2007; Geiser, 2009). However, the final model for an LCA (i.e., how many classes there are) was chosen based on a mixture of statistical indicators and extant theoretical considerations (Nylund et al., 2007). Missing values were expected to be missing at random and therefore estimated using the Full Information Maximum Likelihood (FIML) method integrated in the Mplus LCA-approach (Muthén and Muthén, 2017).

From the class reports the teachers provided, participants’ assigned sex was listed in the traditional dichotomous way. This assigned sex classification was then used as a starting point to explore and develop the intended multidimensional GISA patterns.

## Results

### Results on the First LCA Step: Grouping Students Into Empirically Distinct GISA Patterns According to Four Dimensions

Based on the four dimensions described above (gender identity diversity, gender typicality at school, gender expectations and sexual attraction), we applied LCA in order to group students into empirically distinct GISA “classes” (or patterns). Then, we conducted invariance analysis to ensure reliability for the identified number of patterns and establish the same relevance of the four dimensions for assigned females and males. We carried out these procedures as follows:

First, based on the four dimensions, we tested for invariance (Olivera-Aguilar and Rikoon, 2018) in the number of GISA patterns (configural invariance) that could be analyzed for assigned females and males. We also tested whether the factor loadings were invariant, thus ensuring that the factors’ structures—that is, the four dimensions—were the same for both assigned females and males (metric invariance).

The differences between the BIC scores for the two, three, four, and five class solutions were small, which suggested weak evidence (Raftery, 1995) for a higher class-number solution. Additionally, the results of the two-classes solution were not trustworthy. A BIC rise (ΔBIC = 29) was displayed between classes 3 and 4, and the entropy drop was noticeable from the four-class to the five-class solution. Given the above-reported criteria and the rule of deference to more constrained and parsimonious models (Nylund et al., 2007), the three-class solution was selected.

When testing for metric measurement invariance, we identified a non-significant likelihood ratio chi-square difference test (Δchi^2^[12] = 5.68, *p* > 0.05), thereby establishing the same relevance for the four dimensions for both assigned females and males. Ensuring metric invariance was the first approach necessary for comparing the four dimensions to identify GISA patterns.

In summing up the invariance testing results, we found that the same number of gender patterns and gender dimensions were present across both assigned females and males. In terms of content, that means the four introduced and empirically analyzed dimensions provided an empirically reliable measure for three GISA patterns each for assigned females and males, thus indicating a notable gender heterogeneity for both *cis*-gender heterosexual groups.

### Results on the Second LCA Step: Testing the Connection Between the Three Empirically Validated GISA Patterns and the Adolescent Participants’ Psychological States

The statistical analyses for this procedure with all 11 indicators (four GISA dimensions and seven psychological states) were applied in two sub-steps: Firstly, we ran an LCA separately for assigned females (*n* = 375) and assigned males (*n* = 410) to identify the number of classes and the GISA dimensions’ relevance for an optimal solution when including all 11 reported indicators. Secondly, after establishing separately the same pattern structure for both groups (assigned females and males) we tested the multigroup LCA model in one pooled sample consisting of *N* = 785 adolescents.

In a first step, the LCA models were defined separately for each subsample of assigned females and males (see Supplementary Appendix Table 1), and in a second step in a multi-group model (see Supplementary Appendix Table 2). A detailed description can be found in the Supplementary Appendix.

Referring to the results in the following section, we do not address the scores for each single outcome (see Table 1), but to provide more comprehensible insights, we introduce the LCA and variance analysis results (see Table 1) together. Following our line of reasoning, we introduce the results by specifically displaying if the statistically validated GISA patterns showed variety within a GISA categorization that would remain hidden if we understood gender identity and sexual attraction in a simplistic, dichotomous way.

**TABLE 1:** Variance analysis with Games–Howell *post hoc* test of the 11 analyzed LCA variables in the six patterns (three classes solution).

Classes			1	2	3	4	5	6

Identified gender pattern			Intermediate GISA diverse (assigned females)	Low GISA diverse (assigned males)	High GISA diverse (assigned females)	High GISA diverse (assigned males)	Low GISA diverse (assigned females)	Intermediate GISA diverse (assigned males)

Variables	Welch F asymptotic	η^2^	M (SD)	M (SD)	M (SD)	M (SD)	M (SD)	M (SD)
Gender identity diversity	120.809^***^	44.2	1.22 (0.41)^2,3,4,5,6^	1.06 (0.24)^1,3,4^	2.00 (0.01)^1,2,5,6^	2.00 (0.01)^1,2,5,6^	1.05 (0.23)^1,3,4^	1.03 (18)^1,3,4,5^
Gender performativity at school	57.054^***^	27.5	1.47 (0.50)^2,3,4,5,6^	1.12 (0.32)^1,3,4^	2.00 (0.01)^1,2,5,6^	1.82 (0.38)^1,2,5,6^	1.08 (0.28)^1,3,4,6^	1.22 (41)^1,3,4,5^
Sexual attraction	24.406^***^	10.8	1.37 (0.48)^2,4,5^	1.23 (0.43)^1,3,4^	1.64 (0.48) ^2,5,6^	1.88 (0.32) ^1,2,5,6^	1.20 (0.40) ^1,3,4^	1.22 (42) ^3,4^
Gender expectations	3.409^***^	2.4	1.23 (0.42) ^2^	1.12 (0.32) ^1,6^	1.20 (0.40)	1.17 (0.38)	1.11 (0.32) ^6^	1.25 (43) ^2,5^
Self-esteem	30.318^***^	19.2	1.19 (0.39) ^2,3,5^	1.66 (0.47) ^1,4,6^	1.81 (0.39) ^1,4,6^	1.23 (0.43) ^2,3,5^	1.63 (0.48) ^1,4,6^	1.30 (46) ^2,3,5^
Social competence	10.283^***^	6.8	1.53 (0.50) ^5,6^	1.55 (0.49) ^5,6^	1.61 (0.49)	1.47 (0.51)	1.72 (0.44) ^1,2,6^	1.30 (46) ^1,2,5^
Emotional awareness of one’s emotions	58.944^***^	30.1	1.16 (0.36) ^2,3,5^	1.73 (0.44) ^1,6^	1.58 (0.50) ^1^	1.56 (0.51)	1.83 (0.36) ^1,6^	1.29 (45) ^2,5^
Emotional sensitivity to social context	28.274^***^	18.2	1.28 (0.45) ^2,3,5^	1.67 (0.46) ^1,6^	1.73 (0.44) ^1,6^	1.37 (0.50) ^5^	1.82 (0.38) ^1,4,6^	1.37 (48) ^2,3,5^
Social desirability	5.762^***^	4.5	1.43 (0.49) ^5^	1.56 (0.49) ^6^	1.54 (0.50)	1.52 (0.51)	1.67 (0.46) ^1,6^	1.36 (48) ^2,5^
Dissociation	11.888^***^	44.5	1.73 (0.44) ^2,3,5^	1.06 (0.23) ^1,4,6^	1.17 (0.38) ^1,4,6^	1.65 (0.48) ^2,3,5^	1.13 (0.34) ^1,4,6^	1.77 (41) ^2,3,5^
Fear/depression (Hopkins Scale)	257.241^***^	61.5	1.89 (0.30) ^2,3,5^	1.07 (0.26) ^1,4,6^	1.30 (0.47) ^1,4,6^	1.77 (0.42) ^2,3,5^	1.17 (0.38) ^1,4,6^	1.93 (24) ^2,3,5^

*^1,2,3,4,5,6^Indicate the significant Games–Howell *post hoc* differences between the six classes.*

^***^p < 0.001.

From the three-class solution chosen, we detected a variation on GISA specificity for assigned females and males, such that 8.3% of assigned females (class 3) and 7.1% of assigned males (class 4) displayed significantly higher diversity, which we identified as high GISA diverse (gender diverse/sexual diverse) (see the section “Glossary”). These participants from classes 3 (assigned females) and 4 (assigned males) reported high levels of diverse “gender identity diversity” (e.g., with assigned females stating that they were “more like a boy,” “clearly a boy,” “both” or “none,” and the opposite being reported for assigned males). Assigned males of class 4 and assigned females of class 3 reported higher levels of non-heteronormative typicality at school. For example, assigned males of class 4 reported they expressed themselves as “clearly like girls,” “more like girls,” “both” or “none.” Finally, assigned females and males who identified as “gender diverse” reported higher levels of diverse sexual attraction, meaning they reported being sexually or romantically attracted to people with the same assigned sex “sometimes,” “probably yes,” or “yes” (see Table 1), to “both” or to “none.”

The identified GISA pattern proportions, running the LCA first with just the four GISA dimensions (see Table 2): “high GISA diverse (assigned females)” = 8.1%; “high GISA diverse (assigned males)” = 7.1%) and then the second with the 11 LCA indicators (see Table 3: “high GISA diverse (assigned females)” = 8.3%; “high GISA diverse (assigned males)” = 8.1%), led only to slight deviations. Participants of both “high GISA diverse” adolescent classes (3 and 4) exhibited high levels of being content with their GISA. This holds also for the *cis*/heterosexual (see the section “Glossary”) classes identified, meaning that these adolescents met their own GISA expectations.

**TABLE 2:** Modell fit-indices for a different amount of classes for the multigroup-latent class analysis, configural invariance testing for the four gender dimensions, *N* = 785.

Classes	AIC (dF)	BIC adjusted	Entropy	Remarks
2	−4093 (18)	4120	0.92	Results not trustworthy for one logit threshold
3	−4100 (27)	4140	0.73	
4	−4116 (36)	4169	0.80	
5	−4134 (45)	4201	0.66	

**TABLE 3:** Latent class analysis classification by assigned sex and identified GISA pattern for the chosen LCA-multigroup 3-class solution with all 11 indicators.

Class/gender pattern	Assigned sex	Identified GISA pattern	*N*	% of the pooled sample	% of the respective assigned gender sample
**LCA classification**					
1	Female	Intermediate diverse	202	25.7	53.9
2	Male	Low diverse	238	30.3	57.8
3	Female	High diverse	31	3.9	8.3
4	Male	High diverse	29	3.6	8.1
5	Female	Low diverse	142	18.0	37.9
6	Male	Intermediate diverse	143	18.2	34.8

When checking the pattern variety within the assigned females, we identified the following: when comparing the three assigned females’ classes, 53.9% of the assigned females appeared in the “intermediate GISA diverse (gender identity diverse and/or sexual diverse) class 1 with middle levels of diversity in gender identity; 8.3% of assigned females appeared in the “high GISA diverse (gender diverse/sexual diverse)” class 3; and 37.9% of the assigned females appeared as “low GISA diverse (*cis*/heterosexual)” in class 5. This revealed a clear variation of gender self-perception among assigned females, with class 1 being the most diverse (i.e., gender identity does not coincide with assigned sex and having diverse sexual attraction) and class 5 being the most “low GISA diverse” group (i.e., gender identity matches assigned sex/heterosexual). This shows that in terms of content, just 37.9% of the assigned females (class 5) fit into the “low GISA diverse” profile.

**FIGURE 1:**
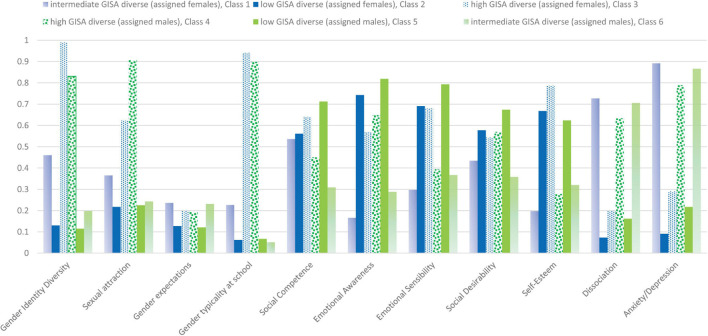
Diagrammatic representation of the identified six gender patterns (three classes solution) by 11 indicators examined by latent class analysis, percentage of category 2 “high level of the specific indicator” in the respective class.

**FIGURE 2:**
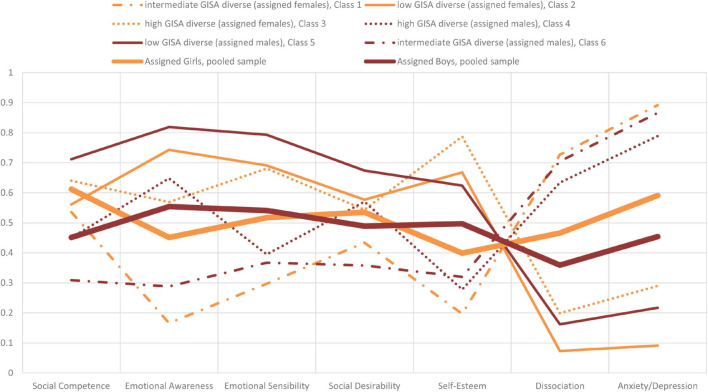
Diagrammatic representation of the six identified GISA patterns plus the two pooled heteronormative gender categories “girls” and “boys” based on the seven indicators examined by latent class analysis, percentage of category 2 “high level of the specific indicator” in the respective class.

As previously mentioned, we found a similar picture when comparing the three assigned males’ patterns with class 4 (being “high GISA diverse”; 8.1% of assigned males), while the assigned males’ GISA pattern class 2 (57.8% of assigned males) considered themselves as mainly “low GISA diverse,” and class 6 (34.8% of the boys) could be considered as “intermediate GISA diverse” with high levels of gender expectations (see Table 1). Interestingly, in comparison to the assigned females (class 5, 37.9%), more assigned males (class 2, 57.8%) considered themselves as cisgender and heterosexual. Notably, 53.9% of assigned females (class 1) were categorized as “intermediate GISA diverse” with middle levels of gender identity diversity and higher levels of diverse sexual attraction. 34.8% of assigned males (class 6) were categorized as “low GISA diverse,” with the same low levels on diversity concerning gender identity and sexual attraction but high levels of gender expectations. The

differences between assigned females and males notwithstanding, we identified a high level of diverse GISA patterns for both assigned sex groups. We further illustrate the distinctions captured in Table 1 with Figures 1, 2 below.

In the next step, we checked to see if the detected six GISA patterns were related in terms of content to different levels of specific psychosocial factors exhibiting more positive or negative symptoms for individual and social development in adolescence (see Table 1 and Figure 1). When compared to the “intermediate GISA diverse” (assigned males) class 6, the assigned males’ “high GISA diverse” class 4 was characterized by high levels of a positive self-concept as self-esteem and emotional awareness, and by higher levels of social competence, emotional sensitivity to others, and social desirability. At the same time, when compared to the “intermediate GISA diverse” (assigned males) class 6, “high GISA diverse” (assigned males) in class 4 had significantly lower levels of dissociation, fear, and depression (Hopkins scale). In sum, individuals in the “high GISA diverse” (assigned males) class 4 expressed a positive psychological state, doing far better than the “intermediate GISA diverse” (assigned males) in class 6. This indicates that the “high GISA diverse” (assigned males) class 4 expressed very positive characteristics when compared to the “intermediate GISA diverse” (assigned males) class 6. Interestingly, the “low GISA diverse” (assigned males) in class 2 had very similar levels on the tested psychosocial factors as those in the “high GISA diverse” (assigned males) class 4, and reported even lower levels on dissociation, fear, and depression (see Table 1 and Figure 1).

The assigned males’ GISA classes’ heterogeneity was also evident when comparing the two assigned males’ classes 2 and 6: When compared to the “intermediate GISA diverse” class 6, “low GISA diverse” class 2 reported higher levels of self-esteem and emotional awareness, and higher levels of social competence and emotional sensitivity to others, while having significantly lower levels on dissociation, fear, and depression (Hopkins scale; see Table 1). Our data showed that the adolescents in the “intermediate GISA diverse” (assigned males) class 6 were more troubled and under significantly more pressure when compared to the respondents in both other classes. The adolescents in the “intermediate GISA diverse” (assigned males) class expressed the lowest levels of positive and highest levels of negative psychological characteristics while those who identified as “high GISA diverse” (assigned males) or “low GISA diverse” (assigned males) were largely free of psychosocial trouble at this age. Notably, not being able to fulfill one’s own GISA expectations was highly demanding for these “intermediate GISA diverse” assigned males. Thus, in sum, when comparing the three identified assigned males’ GISA classes, we found significant differences in gender typicality at school, GISA expectation, and psychological state of mind, with the “intermediate GISA diverse” (assigned males) (class 6) being the most burdened.

The three identified assigned females’ classes — class 1, “intermediate GISA diverse” (53.9%); class 3, “high GISA diverse” (8.3%); and class 5, “low GISA diverse” (37.9%) — each expressed very specific GISA and psychological state profiles and evidenced a notable heterogeneity within assigned females GISA group. Our analysis showed that, as with the “intermediate GISA diverse” (assigned males), the “intermediate GISA diverse” pattern (class 1), 53.9% of assigned females) had the most troubled position when considering their psychosocial characteristics (see Table 1). Assigned females in class 1 (intermediate GISA diverse) reported lower levels of emotional awareness and emotional sensitivity to others, while also reporting significantly higher levels of dissociation, fear, and depression (Hopkins Scale) than the respondents in classes 3 [high GISA diverse (assigned females)] and class 5 [low GISA diverse (assigned females)]. Additionally, individuals in class 1 [intermediate GISA diverse (assigned females)] reported lower levels of social competence and social desirability than those in class 5, [low GISA diverse (assigned females)] but not than those in class 3 (high GISA diverse (assigned females).

In sum, when comparing the three identified assigned females’ GISA classes, we found significant differences in GISA patterns and psychological states of mind, for those in the “intermediate GISA diverse” class 1 (53.9%). Participants in this class expressed a less-than-positive level with regard to their psychological states. At the same time, we also detected a very positive psychological state for the “low GISA diverse” in class 5 (37.9%) and “high GISA diverse” in class 3 (8.3%; see Table 1).

When comparing the explained variance (see Table 1) of the four GISA dimensions for predicting the categorization into the identified GISA patterns, we detected very high levels of “gender identity diversity” (η^2^ = 44.2%) and “gender typicality at school” (η^2^ = 27.5%), and lower but still highly significant levels of “sexual attraction” (η^2^ = 10.8%) and “gender expectations” (η^2^ = 2.4%). This confirmed our theoretical assumptions that gender identity is primarily a question of self-affirmation and typicality, and less, but still intersectionally speaking, a question of sexual attraction.

While the two most diverse GISA patterns, one for assigned males (high GISA diverse, class 4) and one for assigned females (high GISA diverse, class 3), did not differ in their levels of GISA diversity (gender identity diversity, gender typicality at school, sexual attraction, and gender expectations), they differed significantly in their respective levels of self-esteem. The individuals in the “high GISA diverse” (assigned females) GISA pattern (class 3) reported higher levels of self-esteem than the assigned males in high GISA diverse” (assigned males) GISA pattern (class 4; see Table 1). The assigned females’ class 3 (high GISA diverse) also displayed lower levels of dissociation, fear, and depression than the respective assigned males in class 4 (high GISA diverse). At the same time, adolescents in class 3 and 4 reported similar levels of social competence, emotional awareness, emotional sensitivity, and social desirability. Overall, the adolescents in class 3 [high GISA diverse (assigned females)] reported higher levels of a positive psychological state and higher levels of psychosocial well-being than the adolescents in class 4 [high GISA diverse (assigned males)].

Finally, to illustrate the GISA heterogeneity the chosen analytic strategy we compared the levels of category 2 “high level of the specific indicator” of the six GISA patterns on the psychosocial factors to the levels of assigned females and males (see Figure 2). By applying the introduced consecutive LCA analyses according to the intracategorical approach, a new, more advanced empirical model for identifying heterogeneity within GISA emerged.

For example, see in Figure 2 the levels of fear/depression (Hopkins-scale) of the heteronormative categories of girls and boys. So far, the known research (Hyde et al., 2019) has suggested that, when sex is assigned at birth, girls have higher levels (59.1%, posterior probability) of fear/depression than boys do (45.4%, posterior probability). In contrast, via our new GISA model (see Figure 2), we found similarly high levels of fear/depression for “intermediate GISA diverse” (assigned females) (89.2%, posterior probability) and “intermediate GISA diverse” (assigned males) (86.6%, posterior probability), and for “high GISA diverse” (assigned males) (78.9%, posterior probability). Additionally (see Figure 2), “high GISA diverse” (assigned females) reported similarly (29.0%, posterior probability) high levels of anxiety/depression as low GISA diverse (assigned males) (21.7%, posterior probability), while low GISA diverse (assigned males) (9.1%, posterior probability) had the lowest percentage of the high level for fear/depression in comparison to all other groups.

## Discussion

By applying intracategorical intersectionality as a theoretical and critical framework for our study, we had to reshape categories for gender identity and sexual attraction and verify their validity empirically with complex statistical tools. We operationalized our approach by following the broadly applied and multidimensional concept of gender identity Egan and Perry (2001) which we developed further by adapting an intersectionally informed gender-continuum approach instead of a binary gender categorization. We also extended the multidimensional model found in the current literature (Leaper et al., 2012; Kornienko et al., 2016; Martin et al., 2017; Hoffman et al., 2019). With this extension, we did not just take a step forward toward more fluid gender categorizations (Ainsworth, 2015; Richards et al., 2016; Zhao, 2018), we also developed and validated the notion that gender identity and sexual attraction patterns following Butler’s (1999a,b, 2009) critique on the “over-sexualization” of gender where sexuality is certainly intersectionally linked, but by far not a gender category.

The results of our multistep LCA show that the gender binary is far too simplistic when it comes to fully understanding adolescents’ differences and similarities in terms of gender identity and sexual attraction. Out of the subsample of *n* = 785 adolescents (375 assigned females and 410 assigned males), three significant subgroups of multidimensional GISA patterns emerged for assigned females and males, where differences within each assigned sex group were larger than the differences between the assigned sex groups. Our consecutive LCA applications showed that three classes each for assigned females and males could be described as “low GISA diverse (*cis*/heterosexual),” “intermediate GISA diverse (gender identity diverse and/or sexual diverse)” and “high GISA diverse (gender diverse/sexual diverse). The identification of significant membership in the “intermediate GISA diverse” (assigned females) class contradicts Egan and Perry’s (2001) consideration that “knowledge of membership in a gender category” is constant at the age of 6 or 7 years, and is therefore not suitable for measuring within-gender differences. Similarly, the “intermediate GISA diverse” (assigned males) class is facing certain identity difficulties due to unfulfilled GISA-expectations.

Further, the identified GISA variations within the heteronormative categorization of girls and boys are noteworthy. While just about one third of assigned females and approximately every second assigned male identified as fitting into the traditional “low GISA diverse” profile, we also found that about 8% of assigned females and males identified as “high GISA diverse,” and a large number of participants, more than half of the assigned females identified as “intermediate GISA diverse” and more than one third of the assigned males, identified as “intermediate GISA diverse.” That finding alone belies the notion that knowledge of GISA membership is constant by the age of 6 or 7. It also points to the need to allow for gender fluidity in adolescence and before. When approximately only every third assigned female and every second assigned male identified as fitting into the classic *cis*/heterosexual profile, what happens to the others?

The identified GISA patterns expressed very singular GISA and psychological state profiles and thereby a notable heterogeneity within the heteronormative gender groups of girls or boys. Furthermore we showed that for assigned females and males, those found in the “intermediate GISA diverse” groups were in the most troubled position (Eisenberg et al., 2017) when considering their psychosocial state, in that they reported experiencing lower levels of emotional awareness, social competence, and emotional sensitivity to others, while also reporting significantly higher levels of dissociation, fear, and depression than both assigned sex groups in the “high GISA diverse” and “low GISA diverse” classes. We suggest this calls for a well-tuned understanding that for a sizeable number of adolescents, GISA is not fixed, so questioning and diversity needs to be accepted and supported. The levels of assigned females and males did not correspond with the levels identified when split into heteronormative categories of girls and boys. The psychological reality identified via the new GISA categorization gives an almost completely new picture of adolescents’ psychological state, and with that, a more complex identifiable GISA pattern. This new empirical model was statistically confirmed and has proven to be reliable via several statistical steps.

In addition to the challenges science presents, there are enormous costs to maintaining the gender binary. These costs include the myriad negative consequences of gender stereotyping and prejudice. For these reasons, the gender binary should be replaced by a conception of GISA that stresses multiplicity and diversity, including a system whose categories are not mutually exclusive (one can identify as more than one) but are fluid (one’s identity can change across time), and that allows for the possibility that gender identity is irrelevant to the self (Hyde and Mezulis, 2020). In the realm of educational accomplishments, research suggests that gender stereotypes impede children’s achievement in domains culturally viewed as inappropriate for their gender. As early as second grade, children hold the implicit stereotype that math is for assigned males (Cvencek et al., 2011), despite meta-analytic findings that assigned females do as well as assigned males in math. The concern is that, as children and adolescents develop their intentions about a future career, assigned females will not consider careers in math-intensive fields such as engineering and physics because they have absorbed cultural messages that math is for boys, not girls (e.g., Eccles, 1994). In this way, beliefs in the gender binary disempower people and limit human potential.

Interestingly, assigned females and males who identified as “high gender diverse” reported high levels of a positive self-concept, with high levels of self-acceptance and emotional awareness; along with higher levels of social competence and emotional sensitivity to others; and lower levels of dissociation, fear, and depression. This shows that “high GISA diverse” assigned females and males overall expressed very positive characteristics, with, interestingly, very similar levels of the tested psychosocial factors as those that “low GISA diverse” assigned females and males reported. This very positive outcome for the “high GISA diverse” adolescents at age 12.5 is a clear contradiction to the usual view of “high GISA diverse” adolescents as being deficient in terms of psychological state (Boskey, 2014; Clark et al., 2014; Averett, 2016). These positive outcomes at the age of 12.5 and in Grade 7 will have to be tested longitudinally when considering the societal powers that press adolescents into heteronormative GISA categories.

Despite its limitations, the current study has several noteworthy strengths for understanding early adolescents’ GISA patterns. The study’s findings have significant implications for practice and policy by providing information about the heterogeneity of understanding GISA patterns, and about the role of the existing gender regimes on further adolescent development. How gender heteronormativity-enforced social injustice affects adolescents’ lives has yet to be examined. When multilayered gender relationships are shaped by the particular gender regimes of cultural meaning in which adolescents live, they in turn also shape social relations (Mollett and Faria, 2013) as self-imaging. Thus, we have to address the question of societally induced developmental hazards because of hegemonic gender categorization. Given these pressures, we have to ask if the adolescents will just follow the narrow heteronormativity gender path. Will the expected encounters of gender politics’ blunt force, and by that the vulnerability production (Eriksen and Simon, 2017), affect “high GISA diverse,” and “intermediate GISA diverse” adolescents’ psychological stability? If psychological symptoms are societally induced, and homonegativity is internalized (López-Sáez et al., 2020), which terms and processes should we use to describe adolescents’ psychological symptoms when working with higher fear, depression, and suicide levels, given that these are societal, not individual, pathologies (Mills, 2014; White, 2017)?

Based on Butler’s (1999a) concept of “gender performativity,” we connected gender identity and sexual attraction as ongoing sequences of acts and patterns, and placed them in the context of social power discourses. When comparing the explained variance of the our four GISA dimensions (gender identity diversity, gender typicality at school, sexual attraction, and gender expectation) for predicting the categorization into the identified GISA patterns, we detected very high levels of “gender identity diversity” and “gender typicality at school,” and lower but still highly significant levels of “sexual attraction” and “gender expectations.” This supports our theoretical assumptions (Butler, 2009) that gender identity is primarily a question of self-affirmation and typicality, and intersectionally, a question of sexual attraction. Although, there are still possibilities for subversion from within those constraints, even if the notion of “freedom of choice” will always be limited to the discourse within the law or within a given culture.

### Limitations

To reflect on the social power discourses that are the contexts for gender, we applied intersectionality as a way to develop multifaceted and complex GISA patterns for quantitative research. By applying an *intra*sectional and *inter*sectional quantitatively empirical approach, we gained vast insights on the complexity of GISA in adolescence. That said, there is still a need to connect the currently detected GISA patterns to additional intersectional categories such as migration, health, and social status for detecting social intersections when different categories are integrated as a tool to address an even more complex gender realities (McCall, 2005; Westbrook and Saperstein, 2015). By developing this more complex categorization of gender identity, we will face limitations when applying the intercategorical approach because the sample will have to be at least 1,500 students to extend the modeling.

In this study, we analyzed GISA patterns and focused our attention on the gender diversity that pushes gender identity debates and cultural norms on prescribed gender categories, but took into account the adolescents’ young age (12.5) and the possibilities for testing sexual orientation (Savin-Williams and Vrangalova, 2013) because of the individual, social, and biological changes during this period of time (Diamond and Butterworth, 2008; Steensma et al., 2013; Dembroff, 2019). We did not include gender dysphoria as the physical and psychological discomfort caused by the discrepancy between gender identity and assigned sex (Knudson et al., 2010; Fraser, 2015; Poteat et al., 2019). We have to address this issue in future research and fill the gaps with regard to the already analyzed gender diversity. We focused on sexual attraction as one of three components of sexual orientation (specific identity labels, sexual/romantic attraction, and sexual behavior). As adolescents are heterogeneous in how they experience and define their sexual orientation and the three components are not always concordant (Stewart et al., 2019) it would be very interesting for future research to include all three components.

With Butler’s (2009) *Gender Trouble*, we placed gender and sexuality in the context of the social power discourses and therefore rejected gender or “natural” sex essentialism (p. 4). We also acknowledged there is no position of implied freedom beyond discourse and recognized that gender identity and lived sexual attraction are constrained by the power structures within which it is located, but we also acknowledge there are possibilities for subversion from within those constraints, even if the notion of “freedom of choice” will always be limited to the discourse within the law or within a given culture. Generally speaking, in doing this, we don’t abstract ourselves from our bodies, rather we still seek language that allows us to express our embodied gendered and sexual selves.

Furthermore, given the cross-sectional design of our data so far, we cannot state how these GISA patterns will change or evolve during the overall phase of adolescence, and if these possible GISA pattern changes will lead to different self-concept patterns over time (Hyde and Mezulis, 2020). The identified GISA patterns’ stability and their relation to the respective psychosocial indicators during high school (ages 13–16) must be analyzed and can still be called aspirational.

A deeper qualitative understanding of the specific GISA patterns and their meanings for adolescents would be extremely relevant. Case studies would best achieve these insights (Artz, 1998). Using a mixed-methods design is highly recommended for understanding GISA patterns in adolescence in a more future-oriented way. Therefore, sampling, internal and external validity issues, and data collection procedures would have to be reconsidered (Tashakkori and Teddlie, 2010). Different data sources (e.g., teachers, parents, students, and peers), a variety of procedures (e.g., questionnaires, interviews, and observations), and a range of methodologies (e.g., ethnography and experimental approaches) could be combined to deepen the understanding of how GISA is enacted in adolescence.

Additionally, we believe that we need to replicate our model with a larger sample of at least 1,500 students—as the smallest identified groups in our computations were about *n* = 30—to understand specific GISA pattern subgroups and we need to gather this data across more countries to test its validity. In the meantime, we hope to have made a good start with our revision of existing approaches to understanding adolescent students’ GISA patterns. As gender identity is also connected to respective social and societal environments, we still have to find ways to integrate in an empirically valid and reliable way not only self-assessments, but also assessments of social pressures on gender identity, especially for “high GISA diverse” adolescents (Hässler and Eisner, 2020), from significant others (Egan and Perry, 2001) or religious belief systems (Van Droogenbroeck and Spruyt, 2020), and via intergroup-bias (Martin et al., 2017). Additionally, we needed also to explore the possibilities on applying Latent Profile Analysis, instead of LCA, for working with continuous and not only dichotomous categorizations.

In working to further understand the complexity and the fit of sexual attraction within the concept of gender identity, we recognize that there is a need to also include biological components in these models. We therefore need to include biological gender markers, such as measures of steroid hormones such as androstenedione and estrogen to assist in understanding sexual development in puberty so that we can develop a more advanced and bio-psycho-sexual-social understanding of gender identity (Søeborg et al., 2014; Krebs et al., 2019; Merino et al., 2019). Combining the complex factorial net of biological, social, and individual factors concerning gender is a demanding undertaking, and with that comes the additional challenge of understanding what these respective combinations imply for various adolescent groups’ gender development and the call for ongoing research in this burgeoning field.

## Data Availability Statement

The raw data supporting the conclusions of this article will be made available by the authors, without undue reservation, upon completion of the project in 2023.

## Ethics Statement

The studies involving human participants were reviewed and approved by Research Ethics Committee, University of Zurich, Switzerland. Written informed consent to participate in this study was provided by the participants’ legal guardian/next of kin.

## Author Contributions

WK contributed to the leadership and design of the study. WK, DA, and CF contributed to the design of tasks used in the study. DA and CF contributed to the data collection. WK contributed to the data analysis. WK, DA, CF, and SA contributed to the data interpretation. WK contributed to the manuscript authorship. WK, DA, CF, and SA contributed to the critical revision and editing. All authors contributed to the article and approved the submitted version.

## Conflict of Interest

The authors declare that the research was conducted in the absence of any commercial or financial relationships that could be construed as a potential conflict of interest.

## Publisher’s Note

All claims expressed in this article are solely those of the authors and do not necessarily represent those of their affiliated organizations, or those of the publisher, the editors and the reviewers. Any product that may be evaluated in this article, or claim that may be made by its manufacturer, is not guaranteed or endorsed by the publisher.

## Footnotes

1
https://www.sciencedirect.com/topics/medicine-and-dentistry/bayesian-information-criterion

## Glossary

• Assigned sex
  ∘ sex assigned at birth made at an individual’s birth based on an infant’s anatomy and/or sex chromosomes.
• Attraction
  ∘ There are many different types of attraction, including sexual and romantic attraction. Sexual attraction makes people desire sexual contact or show sexual interest in another person(s). Romantic attraction makes people desire romantic contact or interaction with another person or persons. To not experience any sexual attraction toward individuals of any gender is described as asexuality.
• Cisgender
  ∘ we use this term for someone’s sex assigned at birth corresponds to their self-defined gender identity norms.
• Gender
  ∘ is a socially and culturally constructed concept that is developed through experience with the social context.
• Gender diversity
  ∘ when someone’s gender identity does not coincide with their assigned sex. This includes people with no gender identity, transgender and/or non-binary people.
• Gender identity
  ∘ one’s identity within a given society’s understanding of gender.
• Non-binary
  ∘ we use this term to refer to people who don’t subscribe to conventional gender identity categories but identify with both, neither or a combination of gender identities.
• Sexual diverse
  ∘ refers to all the individuals with diversities of sexual orientations, as for lesbians, gays, bisexuals, queers, asexuals and other terms.
• Transgender
  ∘ one’s gender identity and assigned sex do not coincide with societal norms (sometimes this term is used to include non-binary people).
