# Supplementary material for: Multidimensional and Intersectional Gender Identity and Sexual Attraction Patterns of Adolescents for Quantitative Research
Source: Front Psychol. 2021 Sep 17;12:697373. doi: 10.3389/fpsyg.2021.697373 (PMC8485041; doi:10.3389/fpsyg.2021.697373)
Supplement: Supplementary file 1 [file Table_1.docx]

**Appendix**

As for the first step, the LCA models were defined to determine whether there was a significantly differing number of patterns in each subsample of assigned females (n = 375) or assigned males (n = 410). In using LCA to test separately for assigned females and males with all 11 indicators by way of identifying the number of classes and the dimensions’ relevance for an optimal solution, a three-class solution (see Appendix Table 1) was the best one, given a high drop on AIC and BIC in comparison to a two- or four-class solution. Additionally, high entropy and the two tests run (Vuong–Lo–Mendell–Rubin likelihood ratio test and the Lo–Mendell–Rubin adjusted test) indicated the three-class solution as the optimal one. Statistical tests of model fit are in Appendix Table 1. The Vuong–Lo–Mendell–Rubin likelihood ratio test and the Lo–Mendell–Rubin adjusted test both identified the number of classes and were used for the first LCA step for both assigned females and males when running LCA separately.

Appendix Table 1: Modell Fit-Indices for a different amount of classes for Latent Class Analysis with the eleven indicators, run separately for assigned females (n = 375) and assigned boys (n = 410)

| Classes | AIC | BIC adjusted | Entropy | Vuong-Lo-Mendell-Rubin Likelihood Ratio Test, p-value | Lo-Mendell-Rubin Likelihood Ratio adjusted Test, p-value | Remarks |
| --- | --- | --- | --- | --- | --- | --- |
| «assigned females» |  |  |  |  |  |  |
| 2 | 4378 | 4395 | .69 | < .01 | < .01 |  |
| 3 | 4340 | 4366 | .82 | .01 | .01 |  |
| 4 | 4317 | 4353 | .69 | .21 | .21 | Results not trustworthy for three logit thresholds |
| 5 | 4299 | 4344 | .69 | .15 | .15 | Results not trustworthy for five logit thresholds |
| «assigned males» |  |  |  |  |  |  |
| 2 | 4433 | 4452 | .64 | < .01 | < .01 |  |
| 3 | 4391 | 4421 | .76 | .05 | .05 |  |
| 4 | 4364 | 4404 | .75 | .19 | .19 | Results not trustworthy for three logit thresholds |
| 5 | 4341 | 4391 | .78 | .14 | .15 | Results not trustworthy for five logit thresholds |

Secondly, having established the structure and the four GISA dimensions separately for both assigned females and males, we ran the multigroup model with the pooled sample (N = 785 adolescents) comparing assigned females and assigned males simultaneously, again with all 11 indicators (four GISA dimensions and seven psychological states). We selected a latent class multigroup model (see Appendix Table 2) consisting of three classes because it had a lower BIC adjusted score for class 3 (9925) in comparison to a class 2 solution (9968), and the drop to a class 4 solution (9909), or a class 5 solution (9908) was minimal. The differences between the BIC scores for the three-, four-, and five-class solutions were very small, which suggested weak evidence (Raferty, 1995), thus following the law of parsimony, we favored the three-class solution. This was also suggested by the highest log-likelihood drop between the three- and two-class solutions and held in a similar way for the identified entropy as a certainty measure class criterion of estimation because the highest entropy was on the three-class solution. In addition to empirical measures for class determination, we chose the three-class solution as the final model because of ease of class interpretability and theoretical considerations.

Appendix Table 2:

Modell Fit-Indices for a different amount of classes for Latent Class Analysis with all eleven indicators, the multigroup model comparing the two assigned sexes simultaneously, N = 785

| Class | AIC (dF) | BIC adjusted | Entropy |  |
| --- | --- | --- | --- | --- |
| 2 | - 4904 (46) | 9968 | .82 |  |
| 3 | - 4842 (69) | 9925 | .84 |  |
| 4 | - 4794 (92) | 9909 | .78 |  |
| 5 | - 4753 (115) | 9908 | .79 |  |
